# Influence of anesthetic agent and burst suppression on postoperative delirium in elderly patients: a prospective cohort study with automated EEG analysis

**DOI:** 10.3389/fnagi.2025.1743267

**Published:** 2026-01-13

**Authors:** Maximilian Markus, Marc Dorenbeck, Vera Röhr, Sophie Leroy, Benjamin Blankertz, Emery N. Brown, Claudia Spies, Susanne Koch

**Affiliations:** 1 Department of Anaesthesiology and Intensive Care Medicine (CCM/CVK), Freie Universität Berlin and Humboldt Universität zu Berlin, Charité – Universitätsmedizin Berlin, Berlin, Germany; 2 Neurotechnology Group, Technische Universität Berlin, Berlin, Germany; 3 Harvard–MIT Health Sciences and Technology Program, Massachusetts Institute of Technology, Cambridge, MA, United States; 4 Department of Anesthesia, Critical Care and Pain Medicine, Massachusetts General Hospital, Harvard Medical School, Boston, MA, United States

**Keywords:** burst suppression, elderly, general anesthesia, intraoperative EEG, postoperative delirium

## Abstract

**Background:**

Guidelines currently suggest considering EEG guidance during general anesthesia in elderly patients to avoid prolonged burst suppression (BS), with the aim of mitigating postoperative delirium (POD). Our study aimed to investigate the association between POD and intraoperative BS duration dependent on the general anesthetic agent used (propofol vs. sevoflurane).

**Methods:**

In this prospective study (2019–2022), EEGs from 265 patients over 70 years undergoing general anesthesia were analyzed for intraoperative BS duration both visually and using one new automated algorithm to evaluate its accuracy. Associations between BS duration, anesthetic agent, and postoperative delirium (POD) were evaluated using multivariable logistic regression, adjusting for confounders.

**Results:**

BS duration was markedly shorter than in prior cohorts but did not reduce overall postoperative delirium (POD) incidence. POD occurred more frequently with sevoflurane than propofol (44% vs. 30%, *p* = 0.017), despite shorter median BS [0 s (IQR 0–4.9) vs. 20.6 s (IQR 0–151.7); *p* = 0.012]. A significant interaction between anesthetic agent and BS (*p* = 0.033) showed that BS under sevoflurane conferred 3.8-fold greater POD risk than under propofol. Sevoflurane plus BS increased POD odds 9.3-fold compared to propofol without BS. Our new automated BS detection algorithm demonstrated high precision (median error <2.17 s).

**Conclusion:**

Sevoflurane markedly increased POD risk versus propofol, independent of BS duration. Sevoflurane and BS interaction amplified delirium odds. BS appears a vulnerability marker rather than a causal factor. The validated machine-learning BS detector offers a reliable tool for future EEG-based delirium risk research.

## 1 Introduction

Among older patients undergoing anesthesia and surgery, perioperative neurocognitive disorders, including the emergence of postoperative delirium (POD), are widespread, with an incidence of 15–25% in major surgery and up to 50% after high-risk procedures (Heo et al., 2020). In a recent systematic review, the incidence of POD rises with age, affecting up to 84% of older patients with a mean of 23%, when assessed by the Confusion Assessment Method (Jensen et al., 2006), and is more prevalent among those with preexisting cognitive impairments (Bilotta et al., 2021). As the number of elderly individuals undergoing surgical and anesthetic procedures continues to rise, POD has evolved into a substantial global health challenge that requires immediate attention.

As anesthesia deepens, the electroencephalogram (EEG) transitions from a continuous pattern to a characteristic discontinuous pattern of alternating high-amplitude bursts and suppressed low-amplitude activity, a state known as burst suppression (Shanker et al., 2021). Understanding the mechanisms underlying this profound EEG pattern is essential for interpreting what burst suppression signifies about brain state and anesthetic depth:

BS is a profound state of cerebral inactivation characterized by alternating periods of high-voltage activity and electroencephalographic silence. The pathophysiological mechanisms underlying burst-suppression patterns remain incompletely understood, and multiple competing hypotheses have been proposed.

The disconnection hypothesis suggests that BS emerges as an intrinsic cortical rhythm when the neocortex is isolated from thalamic and subcortical afferent inputs, as evidenced by historical observations of BS in surgically isolated cortex and experimental models of pharmacological or anatomical deafferentation (Swank and Watson, 1949; Henry and Scoville, 1952). Steriade et al. (1994) support this by showing thalamic volleys revive silenced cortical networks after prolonged EEG flatness (>30 s), implying “virtually complete disconnection” in thalamocortical circuits drives full-blown BS, with remnant thalamic delta oscillations (1–4 Hz) triggering bursts (Steriade et al., 1994).

The hyperexcitability hypothesis suggests that BS induced by anesthetics like isoflurane, propofol, and barbiturates reflects cortical hyperexcitability due to decreased inhibition from reduced GABA-A receptor activity, leading to elevated interstitial chloride concentrations. Bursts are triggered by subliminal stimuli impinging on this hyperexcitable cortex, while the quasi rhythmic pattern emerges from interplay between hyperexcitability and a Ca2+-dependent postburst refractory period (Kroeger and Amzica, 2007).

The metabolic hypothesis, developed through computational and experimental work by Ching et al. (2012), posits that BS emerges from the interaction between neuronal dynamics and cerebral metabolic rate, wherein ATP-dependent potassium channels play a critical regulatory role.

Critically, the cortex maintains responsiveness during BS states, particularly in the intra-operative environment. Somatosensory stimulation can evoke bursts and alter suppression ratios even during deep BS patterns, indicating that although BS reflects profound depression of spontaneous cortical activity, the cortex retains capacity for stimulus-driven activation (Călin et al., 2014). This reactive property of BS suggests that the pattern reflects both intrinsic neuronal dynamics and preserved afferent responsiveness, complicating the interpretation of what BS signifies about the level of anesthesia or the underlying brain state (Călin et al., 2014).

BS during general anesthesia is referred as a major independent risk factor for POD, with prolonged intraoperative BS duration being associated with higher POD incidence in older surgical patients. A 2024 systematic review and meta-analysis of 3,705 patients found BS presence increased POD relative risk by 41% (22.1% vs. 13.4% incidence), while each additional minute of BS raised odds by ~1.1% (OR 1.011) (Likhvantsev et al., 2024). To identify these patients at risk, intraoperative frontal EEG Monitors have been introduced in the operative setting and are currently suggested by the European Society of Anaesthesiology to reduce the occurrence of BS and thereby mitigate POD (Aldecoa et al., 2024).

Susceptibility to BS increases linearly with age and occurs in both intravenous anesthesia with propofol and inhaled agents such as sevoflurane (Schwerin et al., 2024). This observed effect seemed to be more prominent with propofol, with a probability of BS nearing 1 in the oldest patients, in contrast to 0.6 with sevoflurane (Purdon et al., 2015).

The aim of our study was to investigate the relationship between the duration of intraoperative BS and the incidence of POD by comparing the BS duration between groups of different anesthetic agents used (propofol vs. sevoflurane). Further, we investigated a BS analysis tool developed by our group (Rohr et al., 2022) and benchmarked it against a published BS algorithm.

## 2 Methods

This study investigated perioperative EEG and postoperative delirium in 265 elderly patients (>70 years) enrolled at Charité Universitätsmedizin Berlin between March 2019 and February 2021. Approved by the Charité ethics committee (EA 1/161/17) with informed consent, the study was registered on ClinicalTrials.gov (NCT03879850) and adhered to data privacy regulations.

### 2.1 Inclusion criteria

- Patients aged >70 years
- Planned operation time >1 h
- Expected hospital treatment period of 5 days,
- Anesthesia induction, anesthesia maintenance with either propofol or an inhalative anesthetic agent, such as sevoflurane or desflurane
- The ability to give informed consent

### 2.2 Exclusion criteria

- Patients with a history of neurological disorders or planned neurosurgery
- Patients with psychiatric disorders or current medication of tranquilizers/antidepressants
- Isolation of patients with multi-resistant bacteria
- Inability of the patients to speak and/or read German
- Homelessness or other circumstances where the patient would not be reachable by phone or postal services during follow-up
- Anesthetic protocol other than propofol or sevoflurane for maintenance

### 2.3 POD assessment

Postoperative POD was assessed in the recovery room using the Nursing Delirium Screening Scale (NU-DESC) every 15 min, five times in total. Followed by a twice-daily assessment of POD with the NuDesc, Delirium Detection Score (DDS) and DSM-V Criteria for Delirium on the ward up to postoperative day 5. Patients who were referred to the Intermediate Care Unit or Intensive Care Unit were assessed using the Confusion Assessment Method (CAM-ICU) twice daily up to postoperative day 5. All tests were performed by trained study personnel with individual training on the detection of POD.

### 2.4 EEG data analysis

The EEGs were recorded with a frontal EEG Monitor (SEDline Root monitor© Masimo Corporation, USA) from the beginning of anesthesia induction to the intraoperative setting up until 1 h in the recovery room. We used four electrodes (Fp1, Fp2, F7, and F8) with the earth electrode at Fpz and the reference electrode 1 cm above. The sampling frequency was 178 Hz. The. Impedances were kept under 5 kΩ. For the study, a monitor was employed for the measurement of all subjects. This ensured that all display settings were uniform for the measurements obtained. The raw EEG file was downloaded from the monitor via USB, and further analysis was performed using the Brain Vision Analyzer (Germany).

In this study, bursts were defined according to the American Clinical Neurophysiology Society’s Standardized Critical Care EEG Terminology (2021) as waveforms lasting at least 0.5 s and containing a minimum of 4 phases (polyphasic) (Hirsch et al., 2021; Rampil, 1998). Suppression was defined based on amplitude criteria commonly used in the literature and in BS probability algorithms, with a threshold set at 5 μV to delineate suppressed EEG segments (Chemali et al., 2013). Each epoch of BS was delimited by the first suppression period in the preceding activity and the last suppression period before the resumption of EEG activity. The BS duration was annotated in seconds by an expert and the results resembled a logarithmic distribution. We further converted the BS duration to a logarithmic scale for enhanced visualization and transformed the data into a standard distribution.

### 2.5 Statistical analysis

Statistical analysis was conducted using SPSS © (Version 29) and Python (Version 3.13). Patients were assigned to the POD group if they tested positive on delirium screening on at least one occasion, either within the first hour after surgery or during the first five postoperative days.

We calculated the median BS durations for the POD and NoPOD groups and used the Mann–Whitney *U* test to investigate the differences between the two groups. Next, we separated the patients into propofol and sevoflurane groups and compared the median BS duration between patients with POD and NoPOD within each anesthetic group using the Mann–Whitney-*U*-test.

### 2.6 Multivariable regression analysis

Statistical analysis was conducted using Python (version 3.13) with statsmodels for logistic regression modelling. A comprehensive multivariable logistic regression approach was employed to examine the independent associations between anesthetic agents, burst suppression patterns, and postoperative delirium while controlling for relevant confounders.

#### 2.6.1 Primary analysis

A binary logistic regression model included anesthetic agent (sevoflurane vs. propofol), age, ASA score, surgery duration (hours), burst suppression, and postoperative care location (recovery room vs. PACU/ICU) as predictors of POD.

#### 2.6.2 Burst suppression modelling approaches

Given the highly skewed distribution of burst suppression duration with many zero values, multiple modelling strategies were employed:

1. Continuous BSR: raw burst suppression ratio as a continuous predictor
2. Raw duration model: burst suppression duration in seconds
3. Binary indicator: presence/absence of any burst suppression (BS duration >0)
4. Sensitivity analyses: restricting analyses to patients with measurable burst suppression
5. Subgroup analyses: sevoflurane only
6. Subgroup analyses: propofol only
7. Interaction model

#### 2.6.3 Subgroup analyses

Separate logistic regression models were fitted for sevoflurane and propofol groups to examine anesthetic-specific effects of BS, surgery duration, ASA score, and age on POD risk.

#### 2.6.4 Interaction analysis

To investigate whether the effect of BS on POD differs between anesthetic agents, we tested for statistical interaction by including a product term (sevoflurane × any_BS) in our multivariable logistic regression models. This approach allows assessment of whether the BS-POD relationship is modified by the choice of anesthetic agent.

#### 2.6.5 Sensitivity analyses

Additional models restricted to patients with measurable BS (duration >0) were performed to assess dose–response relationships among those experiencing EEG suppression.

**Figure 1:**
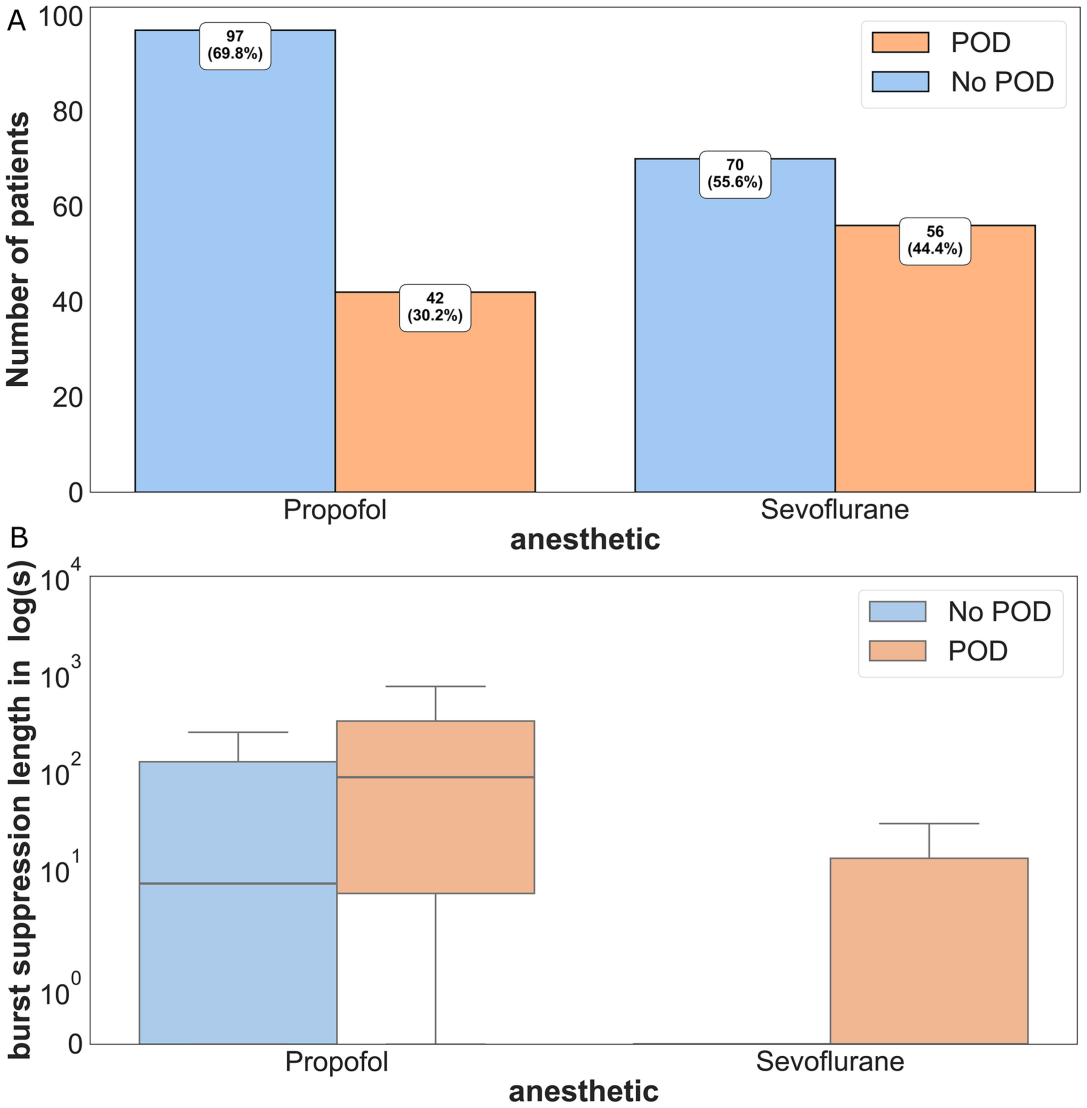
Incidence of POD and no POD for each anesthetic protocol (propofol vs. sevoflurane) in percentage of patients (%). There was a significant statistical difference (*p* = 0.017) between the incidence of POD in the two groups using the Chi-square test. Intraoperative burst suppression duration in propofol and sevoflurane patients, differentiated by POD and no POD, with the ordinate on a logarithmic scale. Propofol patients with POD had a longer mean burst suppression duration compared to patients without POD (POD: 86 s, IQR: 345 s vs. no POD: 7 s, IQR: 124 s, *p* = 0.016). The same effect could be observed for the intraoperative burst suppression duration in sevoflurane patients. In this group, patients with POD had a longer median burst suppression duration compared to patients without POD (POD median: 0 s, IQR: 14 s vs. no POD median: 0 s, IQR: 0 s, *p* < 0.001). POD = Postoperative delirium, No POD = No postoperative delirium.

To evaluate the statistical robustness of our primary finding, a *post-hoc* power analysis was conducted to assess the adequacy of the sample size and determine the achieved statistical power. The analysis aimed to quantify the probability of detecting the observed difference in the incidence of postoperative delirium (POD) between the sevoflurane and propofol anesthetic cohorts. The calculation was performed using G*Power software (Version 3.1) for a *z*-test comparing two independent proportions. The analysis was based on a two-tailed test with a significance level (*α*) set at 0.05. The input parameters included the empirically observed POD incidence rates of 44.4% in the sevoflurane cohort (*n* = 126) and 30.2% in the propofol cohort (*n* = 139), which corresponds to an effect size (Cohen’s *h*) of 0.296. This analysis yielded an achieved power (1−*β*) of 0.84, indicating an 84% probability of successfully detecting an effect of the observed magnitude, which suggests the study was sufficiently powered for this primary endpoint (see Figure 1).

### 2.7 Automated burst suppression analysis

In addition to expert annotations, we evaluated an automated algorithm for BS detection. The algorithm was trained and validated using a 10-fold stratified cross-validation scheme, using the expert annotations as ground truth data separately for each anesthetic group. The algorithm applies amplitude thresholding and windowed averaging to identify suppression phases, with thresholds optimized for each training set. BS was identified only when detected across all EEG channels. Algorithm performance was quantified as the mean absolute error in BS duration or burst suppression ratio (BSR) and optimized based on the lowest mean squared error. Full methodological and technical details are provided in the Supplementary materials.

## 3 Results

### 3.1 Baseline characteristics

In this study, 348 out of 1,237 screened patients were enrolled. After excluding 82 patients due to surgical cancellations/protocol deviations/EEG artefacts, or incomplete data, 265 patients remained for analysis of their raw EEG. For a detailed description (see Table 1; Supplementary material).

**Table 1:** Baseline characteristics of patient cohort.

Variable	Description	NoPOD	POD
		*N* = 167	*N* = 98
Age (years, mean, 95%-CI)		77 (76–78)	77 (76–78)
Sex (*N*, %)	Male	66 (39.5%)	49 (50.0%)
	Female	101 (60.5%)	49 (50.0%)
BMI (kg/m^2^, mean, 95%-CI)		25 (23–26)	26 (25–27)
ASA-score (*N*, %)	1	5 (3.0%)	3 (3.1%)
	2	75 (44.9%)	42 (42.9%)
	3	86 (51.5%)	49 (50.0%)
	4	1 (0.6%)	4 (4.1%)
Highest education (*N*, %)	9th grade leaving certificate	19 (12.8%)	17 (20.7%)
	Intermediate school leaving certificate	45 (30.2%)	29 (35.4%)
	Higher education entrance qualification, master craftsmen	31 (20.8%)	13 (15.9%)
	Bachelor’s degree	9 (6%)	5 (6.1%)
	Master’s degree, university diploma or higher	45 (30.2%)	18 (22.0%)
Surgery type (*N*, %)	Ophthalmology	1 (0.6%)	0 (0.0%)
	Traumatology/orthopedics	40 (24.0%)	25 (25.5%)
	ENT/oral and maxillofacial surgery	4 (2.4%)	2 (2.0%)
	Thoracic surgery	6 (3.6%)	5 (5.1%)
	General surgery	70 (41.9%)	50 (51.0%)
	Gynecology	36 (21.6%)	8 (8.2%)
	Other	10 (6%)	8 (8.2%)
Premedication (*N*, %)	No premedication	159 (95.2%)	96 (98.0%)
	Midazolam p.o.	8 (4.8%)	2 (2.0%)
	Midazolam i.v.	0 (0.0%)	0 (0.0%)
Surgery duration (min, mean, 95%-CI)		149 (132–166)	198 (176–221)
Anesthesia maintenance (*N*, %)	Propofol	97 (58.1%)	42 (42.9%)
	Dose mg/kg/h (95% CI)	6 (6–6)	5 (5–6)
	Sevoflurane	70 (41.9%)	56 (57.1%)
	Endtidal Sevo %	2 (2–2)	2 (2–2)
Postoperative transfer (*N*, %)	Recovery room	87 (52.1%)	43 (43.9%)
	ICU	63 (37.7%)	45 (45.9%)
	PACU	17 (10.2%)	10 (10.2%)

This table shows the baseline characteristics for our patient cohort. POD, postoperative delirium; NoPOD, no postoperative delirium.

### 3.2 Overall incidence of postoperative delirium

Postoperative delirium (POD) developed in 98 patients (37%). POD incidence was significantly higher in the sevoflurane group compared to propofol (56/126, 44% vs. 42/139, 30%; *p* = 0.017, RR 1.36, 95% CI: 1.04–1.76).

### 3.3 Association of burst-suppression duration and postoperative delirium

Overall, patients with POD had longer median burst suppression durations than those without POD [6.95 s (IQR: 0–92.97 s) vs. 0 s (IQR: 0–48.99 s), *p* = 0.010]. However, sevoflurane patients experienced significantly shorter BS durations than propofol patients [0 s (IQR: 0–4.94 s) vs. 20.64 s (IQR: 0–151.71 s), *p* = 0.012], despite having higher POD rates.

### 3.4 Multivariable regression analysis

#### 3.4.1 Primary predictors of POD

In the comprehensive multivariable model (*n* = 262), anesthetic agent was the strongest independent predictor of postoperative delirium. Patients receiving sevoflurane had 3.28-fold higher odds of developing POD compared to propofol recipients (OR 3.27, 95% CI: 1.73–6.24, *p* < 0.001) after adjusting for all other variables (see Supplementary material).

#### 3.4.2 Burst suppression effects

The presence of any intraoperative burst suppression (binary indicator) was significantly associated with POD (OR 2.99, 95% CI: 1.61–5.58, *p* = 0.001). Notably, binary presence/absence of burst suppression was more predictive than continuous duration (OR 1.00, 95% CI: 0.41–1.43, *p* = 0.50) or ratio measures, with continuous BSR (OR 1.04, 95% CI: 0.97–1.11, *p* = 0.257).

##### 3.4.2.1 Surgery duration

Longer operations independently increased POD risk, with each additional hour associated with 27% higher odds (OR 1.27, 95% CI: 1.08–1.51, *p* = 0.005).

##### 3.4.2.2 Non-significant variables

Age (OR 0.997, 95% CI: 0.94–1.06, *p* = 0.923), ASA score (OR 1.02, 95% CI: 0.64–1.63, *p* = 0.939), and postoperative care location showed no independent association with POD.

### 3.5 Anesthetic-specific subgroup effects

Sevoflurane group (*n* = 123): any burst suppression was strongly predictive (OR 5.98, 95% CI: 2.41–14.85, *p* < 0.001), and surgery duration remained significant (OR 1.41 per hour, 95% CI: 1.12–1.77, *p* = 0.003).

Propofol group (*n* = 139): neither burst suppression (OR 1.71, 95% CI: 0.71–4.13, *p* = 0.227) nor surgery duration (OR 1.13 per hour, *p* = 0.211) reached statistical significance.

### 3.6 Anesthetic agent-burst suppression interaction

A statistically significant interaction was observed between the anesthetic agent and the presence of burst suppression. This interaction term (OR 3.80, 95% CI: 1.11–13.01, *p* = 0.033) indicates that the effect of burst suppression on the POD is fundamentally different between the two anesthetic agents.

In the presence of this significant interaction, the main effects on POD risk should be interpreted conditionally.

- Propofol without burst suppression: Baseline risk (OR = 1.00).
- Propofol with burst suppression: Moderate risk increase (OR = 1.50; 95% CI: 0.63–3.55).
- Sevoflurane without burst suppression: moderate risk increase (OR = 1.63; 95% CI: 0.69–3.89).
- Sevoflurane with burst suppression: Dramatic risk increase (OR = 9.30; 95% CI: 1.64–52.76).

These differing results in the interaction model show that although the occurrence of burst suppression episodes is associated overall with the occurrence of a POD, the greater effect in terms of the risk of a POD is associated with the use of sevoflurane.

Among patients experiencing intraoperative burst suppression, those anesthetized with sevoflurane demonstrated 6.2-fold higher odds of postoperative delirium compared to those receiving propofol.

### 3.7 Algorithm performance

**Figure 2:**
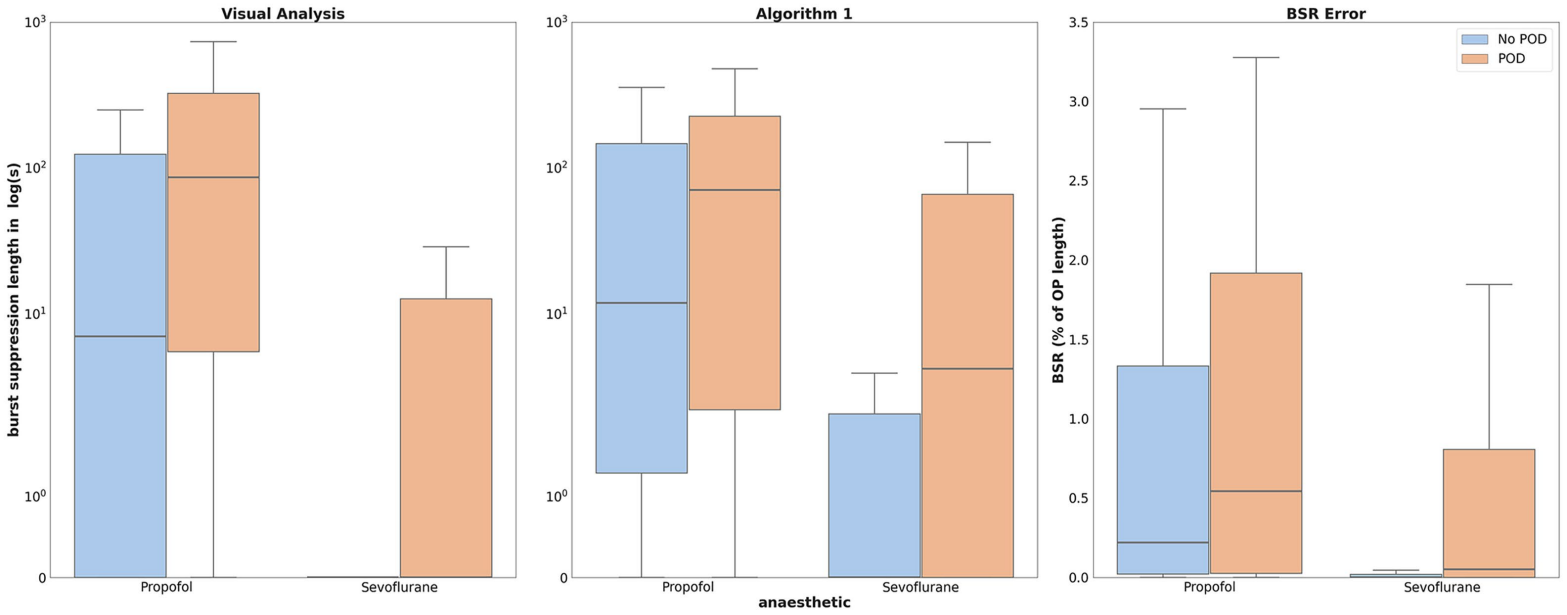
Intraoperative burst suppression duration in propofol and sevoflurane patients, differentiated by POD and No POD, with the ordinate on a logarithmic scale. The visual analysis is compared to the algorithm used for the BS detection. The median error for propofol is 0.13% for POD and 0.1% for NoPOD. For sevoflurane the median error is 0.01% for POD and 0% for NoPOD.

The algorithm demonstrated consistent and reliable detection of BS compared to the visually annotated results. The median error in seconds for the algorithm was 2.17 s [IQR: 0–23 s]. These findings were based on the entire cohort (see Figure 2).

When adjusting for the length of surgery, the median error in the BSR was below 0.1% [IQR 0–0.36%]. The algorithm performed better in the sevoflurane cohort (see Figure 2).

The performance of the algorithm depends on the chosen threshold (see Supplementary material Section 1). It is notable, however, that within the given parameter range, the median BSR error does not exceed 0.1% (Supplementary Figure 2).

## 4 Discussion

Our interaction model reveals that the relationship between BS and postoperative delirium is fundamentally different depending on the anesthetic agent used. The statistically significant interaction between anesthetic agent and BS (OR 3.80, 95% CI: 1.11–13.01, *p* = 0.033) demonstrates risk profiles. This interaction indicates that BS episodes are approximately 3.8 times more deleterious when occurring under sevoflurane compared to propofol anesthesia. Most remarkably, despite achieving burst suppression durations as low as in the second range (measured in seconds per episode), representing a substantial improvement from earlier studies where BS durations were typically reported in the minute range, POD incidence remained high at 44% in the sevoflurane group versus 30% in the propofol group (Soehle et al., 2015). These findings suggest that not the BS duration alone but the combination of burst suppression with the anesthetic drug determines the POD risk.

Additionally, we investigated a new BS detection model, demonstrating a high degree of agreement with the gold-standard visual analysis of EEG recordings, further supporting the robustness of our findings and facilitating further research in this area.

### 4.1 Burst suppression duration and its impact on postoperative delirium

Recent ESA guidelines suggest processed EEG monitoring may help reduce POD (Aldecoa et al., 2024). Soehle et al. (2015) found that longer intraoperative BS durations were associated with higher POD risk in cardiac surgery patients. In contrast, our study observed much shorter BS durations, measured in the second range rather than in the minute range, yet the POD incidence remained high at 37%. This is consistent with the ENGAGES and ADAPT-2 trials, which also found that reducing BS duration did not lower POD incidence (Wildes et al., 2019; Tang et al., 2020).

These findings indicate that preexisting brain vulnerability increases the risk of both BS and POD. Patients with greater sensitivity to volatile anesthetics experience more BS and higher POD rates, even at lower anesthetic concentrations (Fritz et al., 2016). Our recent EEG analysis showed that preoperative EEG could predict POD risk with over 70% accuracy, independent of intraoperative BS duration (Pollak et al., 2024).

### 4.2 Differences in anesthetic protocol

Cao et al. found that propofol reduced the incidence of postoperative delirium (POD) by one-third compared to sevoflurane, while Koch et al. (2023) and Yoon et al. (2012) reported shorter BS durations with sevoflurane, despite higher POD rates with inhaled anesthetics (Cao et al., 2023). Perioperative neuroinflammation, driven by cytokines such as TNF-α, is increasingly recognized as a key factor in POD (Aldecoa et al., 2024; Terrando et al., 2010; Xu et al., 2023; Guan et al., 2023). Animal studies suggest sevoflurane may exacerbate neuroinflammation and cognitive dysfunction, whereas propofol appears to reduce inflammatory responses and protect against cognitive deficits (Dai et al., 2021; Mitsui et al., 2022).

In our study, patients receiving sevoflurane were more likely to develop POD even though they experienced shorter periods of BS. These differences are likely rooted in the distinct mechanisms of action of the two anesthetics. Propofol is a GABA_A_ agonist that tends to induce BS, whereas sevoflurane has GABAergic activation and blocks potassium channels, HCN channels, and NMDA receptors (Hemmings et al., 2005). These differences manifest in varying EEG signatures: propofol induces coherent alpha and slow-delta band power, whereas sevoflurane induces coherent theta power (Purdon et al., 2015; Akeju et al., 2014).

### 4.3 Mechanistic implications of the anesthetic-burst suppression interaction

The significant interaction between anesthetic agent and burst suppression provides distinct insights into POD pathophysiology. This 3.8-fold interaction effect indicates that sevoflurane-induced BS reflects a more vulnerable brain state compared to propofol-induced suppression. This supports the concept that burst suppression is not merely a marker of anesthetic depth, but rather a manifestation of agent-specific neuronal vulnerability patterns.

The interaction analysis reveals that patients receiving sevoflurane who experience any intraoperative BS face a 9.3-fold increased risk of POD compared to baseline, while propofol patients with BS show only a moderate 1.5-fold risk increase. This differential risk profile has implications for perioperative risk assessment and anesthetic management decisions in elderly patients.

These findings elegantly resolve the apparent paradox observed in our primary analysis: despite propofol causing longer BS durations, sevoflurane was associated with higher POD rates. The interaction analysis demonstrates that the clinical significance of BS is context-dependent, with similar EEG patterns carrying vastly different prognostic implications depending on the underlying anesthetic mechanism.

This context-dependency suggests that future EEG monitoring algorithms should incorporate anesthetic-specific risk weighting rather than treating all BS episodes equivalently.

### 4.4 Confirmation of visual examination by automated analysis

The standard for detecting BS activity in EEG recordings is a visual analysis of the raw EEG file, identifying isoelectric EEG lines below a 0.5 μV amplitude over a period of more than 1 s (Rampil, 1998). However, most anesthesiologic studies have used built-in automated BS detection and BSR calculation from EEG monitors as a method of quantifying BS in intraoperative EEG measurements (Soehle et al., 2015; Fritz et al., 2016). For example, the bispectral index (BIS) detects BS when the amplitude falls below a limit of 5 μV for a duration of more than 0.5 s (Rampil, 1998). Similarly, the Cerebral State Index (CSI) detects BS when the signal amplitude falls below 3.5 μV (Jensen et al., 2006). Existing automated BS detection, however, may not be sufficiently accurate, as Muhlhofer et al. pointed out (Muhlhofer et al., 2017). Their study showed that automated BSR analysis employed by Masimo SedLine devices significantly underestimated the BS duration when compared to visual analysis (Muhlhofer et al., 2017). Furthermore, Fleischmann et al. showed that there are significant differences in BS patterns between different anesthetic agents and that these differences may impede accurate data analysis using automated BS algorithms if not considered (Fleischmann et al., 2018). Hence, we performed a visual analysis of our EEG Data and confirmed it using a new algorithm.

The algorithm was trained on a small subset of the labelled data for each of the 10 folds. They showed that the results were consistent with the visual analysis. For future analysis of big datasets, it is therefore sufficient to label a small subset of patients for BS to estimate the algorithm parameters and then use either algorithm on the full dataset.

### 4.5 Limitations

Our analysis of the synergistic interaction between sevoflurane and BS on postoperative delirium risk is restricted to elderly patients (≥70 years). Whether this interaction effect persists, is attenuated, or is absent in younger surgical populations remains unknown and cannot be inferred from our data. Our patient cohort was selected from a university hospital to include older and more severely ill patients who met the specified inclusion criteria. Therefore, these results cannot be generalized to the broader population. The administered drugs focused on the maintenance phase. During the induction phase, it is possible that the patients who received propofol or thiopental for the induction of anesthesia were later classified according to the sedation-maintenance drug. Additionally, BS occurring specifically during the induction phase was not quantified or analyzed as a distinct exposure variable, representing a limitation of our current dataset. It is assumed that the anesthetic administered for induction had already ceased after ~15 min and therefore had a limited effect on the occurrence of intraoperative BS activity and by the time of emergence from anesthesia on the occurrence of POD. The non-randomized assignment of anesthetic agents may have introduced selection bias. We therefore added available confounders to the multivariable modelling and interaction analysis to account for this. POD assessment relied on screening tools with known performance characteristics but potential for false positives during early recovery (emergence agitation). While multi-tool assessment by trained personnel minimized misclassification, Our POD rate may reflect a vigilant detection, even though our POD rate of 37% is in the range of POD in an elderly cohort of >70 years.

Despite finding a strong main effect of anesthetic agent on post-operative delirium risk, our dose–response analyses revealed no significant relationship between anesthetic dose and delirium incidence within either the sevoflurane or propofol groups. This absence of traditional dose-dependent effects, despite clear differences in agent-related POD risk, suggests that individual variability in preexisting brain vulnerability and hence different sensitivity to anesthesia may be more important than absolute drug dosing in determining delirium outcomes. We acknowledge that heterogeneity in patient pharmacokinetics, underlying cognitive reserve, cerebrovascular autoregulation, and baseline neuroinflammatory status likely creates highly variable relationships between drug concentration and neuronal effects across our patient population. The dose-dependent variability in individual brain vulnerability to BS and POD may therefore mask any population-level dose–response trend. BSR as a continuous measure was not significantly associated with POD in multivariable models, unlike the binary presence/absence of BS. This discrepancy likely reflects the extremely low BSR values (<1%) and brief episodes (seconds) in our cohort, which dilute quantitative associations when averaged over long surgeries. The known cortical reactivity during anesthetic-induced BS, where surgical stimuli may evoke bursts and interrupt suppression, may further attenuate BSR’s predictive power. Our analysis employed POD as a binary outcome (presence vs. absence of POD), which precluded evaluation of delirium recovery trajectories, temporal burden, or cumulative cognitive impact across the postoperative period. The anesthetic agents may differ not only in POD incidence but also in the timing, severity, and resolution of POD episodes. Future studies incorporating longitudinal POD burden metrics would clarify whether observed differences in POD incidence translate to clinically differences in recovery dynamics.

### 4.6 Conclusion

Our EEG analysis model precision proved to be reliable, showing very high correlation with visual EEG assessments. The significantly different risk of POD for patients with a BS pattern depending on the anesthetic used underscores the importance of choosing wisely the anesthetic, especially for older patients. Identical EEG pattern (BS) have markedly different prognostic significance depending on the anesthetic administered. Overall, the use of sevoflurane was associated with a higher risk of POD, with this effect being dramatically increased in the presence of BS (9.3-fold increase in risk). These results suggest that BS must be evaluated in the context of the agent-related neural networks it triggers, as it is more likely to be an indication of pre-existing brain vulnerability rather than an equivalent marker of the depth of anesthesia. This supports the need for the development of precision anesthesia approaches tailored to the individual risk profiles.

## Data Availability

The raw data supporting the conclusions of this article will be made available by the authors, without undue reservation.

## Author’s note

This work has been presented as an abstract at the Euroanesthesia Congress on 25.05.2025 in Lisbon.
