# Supplementary material for: Influence of anesthetic agent and burst suppression on postoperative delirium in elderly patients: a prospective cohort study with automated EEG analysis
Source: Front Aging Neurosci. 2026 Jan 13;17:1743267. doi: 10.3389/fnagi.2025.1743267 (PMC12835323; doi:10.3389/fnagi.2025.1743267)
Supplement: Supplementary file 1 [file Data_Sheet_1.docx]

Supplementary Material

# **Influence of anesthetic agent and burst suppression on postoperative delirium in elderly patients: a prospective cohort study with automated EEG analysis.**

Authors:

Maximilian Markus^1^, Marc Dorenbeck^1^, Vera Röhr^2^, Sophie Leroy^1^, Benjamin Blankertz^2^, Emery N. Brown^3^, Claudia Spies^1^, Susanne Koch^1,^

**Affiliations**:

^1^Charité – Universitätsmedizin Berlin, Corporate Member of Freie Universität Berlin and Humboldt Universität zu Berlin, Department of Anaesthesiology and Intensive Care Medicine (CCM/CVK), Berlin, Germany

^2^Neurotechnology Group, Technische Universität Berlin, Berlin, Germany

^3^Harvard–MIT Health Sciences and Technology Program, Massachusetts Institute of Technology, Cambridge, Massachusetts; and Department of Anesthesia, Critical Care and Pain Medicine, Massachusetts General Hospital, Harvard Medical School, Boston, Massachusetts

# Description of the Automated Burst Suppression Analysis

In addition to the professional annotations of the BS, we trained two algorithms on a small subset of the data, taking the labelled data as the ground truth and then predicted the suppression phases with both algorithms.

To avoid choosing a specific training test split, we invert a 10-fold stratified cross-validation scheme, where we take a 10th of the data to estimate the algorithm parameters and test it on the rest of the data. Therefore, we estimated the BS for each patient nine times and used the patient to train the model once. The data were stratified based on the presence of BS in the labelled data. The parameters were estimated for each medication group.

**Algorithm 1** uses a simple two-step process to estimate the total suppression and burst suppression ratio (BSR) for comparison with the labelled ground truth ^1, 2^. We first filtered out high-amplitude artefacts and then applied a second-order Butterworth bandpassfilter between 0.3 and 44 Hz.

In the first step of the BS estimation, we evaluated whether a recorded time point is likely a part of the suppression phase for every channel. To this end, we define the threshold T1. T1 was optimized through a parameter search of the training set of data. Possible values for T1 are the mean M of the absolute values of the amplitudes of the signal, and the quantiles of the amplitudes after bandpass filtering are 0.5, 0.55, and 0.6. Any amplitude below T1 is represented by 1, indicating that this time point is more likely part of a suppression phase; the other values are represented by 0. To account for the fact that the BS is a pattern sustained over time, we take a one second window around each time point and calculate the mean over the window for every channel. The mean represents the suppression probability at each time point for each channel.

In the second step, we define the time points that are part of the sustained suppression phase. Any value higher than a second threshold T2 will be categorized as a suppression time point per channel; if the time point is categorized as a suppression time point for all channels, it will count as a suppression time point. T2 is calculated depending on the amplitude mean M to account for patients with a very low mean amplitude who likely have BS. If M is below 1.5, the data are scaled to a 1.5 amplitude mean.

$$T2=0.99-\frac{0.99}{M}$$

We presented the results of Algorithm 1 in the main manuscript, as this algorithm demonstrated superior performance characteristics for clinical burst suppression detection. Algorithm 1 showed higher precision and lower error rates in validation analyses compared to Algorithm 2, particularly when adjusted for surgery duration (median BSR error <0.001). We believe Algorithm 1 is therefore most suitable for future clinical implementation in intraoperative EEG burst suppression detection systems. Algorithm 2 is included in the supplementary material for methodological transparency and to demonstrate consistency of findings across different detection approaches.

As **Algorithm 2**, we reimplemented the algorithm introduced by Westover in 2013^3^. This Algorithm uses the local mean (1) and variance (2) of the data to classify time points as either burst or suppression periods through adaptive variance thresholding.

$\mu_{t}=\beta\mu_{t-1}+\left( 1-\beta\right)x_{t}$(1)

$$\sigma_{t}^{2}=\beta\sigma_{t-1}^{2}+\left( 1-\beta\right)\left( x_{t}-\mu_{t} \right)^{2}\left( 2 \right)$$

$$z_{t}=\delta\left[ \sigma^{2}<\theta\right]\left( 3 \right)$$

where $x_{t}$is the EEG signal amplitude at time point t, β is forgetting parameter (0-1), which controls the locality of the mean and variance by controlling how much weight previous time points have compared to the current. $\mu_{t}$is the local mean of the EEG signal at time t. $\sigma{^{2}}_{t}$is the local variance of the EEG signal at time t. Both are computed recursively. θ is the variance threshold parameter for burst/suppression classification, which has to be set. δ is the binary indicator function, which returns 1 if the condition is true, 0 if it is false. $z_{t}$is the final binary classification output (1 = suppression, 0 = burst)

We maintained the same forgetting time parameter β as specified in the original paper^1^. The threshold θ was determined through parameter search on a training dataset, testing values of 1, 5, 10, and 20.

For both algorithms, the BS time points are calculated for each channel. Finally, we intersect the suppression phases for each channel and only keep the time points as BS time points for each patient, which are BS time points for all channels.

Performance is measured as the absolute error divided by the operation length or absolute BS ratio (BSR) error.

In the parameter search, the parameter with the smallest mean squared error in seconds was chosen to ensure a balance between the small mistakes made for most patients with little BS and potentially large mistakes made for outlier patients with very long suppression phases. Supplementary Figures and Tables

# Parameter choice in the automated burst suppression estimation

Each of the two algorithms takes a parameter that is used as a threshold to define which time points are likely part of a suppression phase. For both algorithms, it is crucial that the data is preprocessed to cut out any high amplitude artefacts and if there is a shift in the data away from a zero mean, to correct for it. The latter is automatically done by the bandpass filter, which also reduces high-frequency noise.

In the following, the error will always be displayed as the burst suppression ratio error or the error in seconds scaled by the full length of the operation. Since the error accumulates over time, a suboptimal threshold that either over- or underestimates suppression phases becomes increasingly problematic during longer operations.

The threshold theta for Algorithm 2 has to be trained on a subset of the corresponding data, since there is no suggested range for it and the error increases fast, when the threshold is not close to the optimal threshold. (Suppl. Figure 2)

The threshold T1 in Algorithm 1 has a simple correlation in the data, it is dependent on the absolute values of the amplitudes. Therefore, it is reasonable that the 0.8^th^ quantile of the data will lead to overestimation of the burst suppression and the 0.3^th^ quantile will lead to underestimation. The suggested range for the threshold T1 is therefore between the median or mean of the absolute values of the amplitude mean and the 0.6^th^ quantile. The error depends on that parameter, but within the given parameter range, the mean BSR error does not exceed 0.009 or 0.9 % of the operation length, which is a mean error of 100 s. (Suppl. Figure 1)

Even if the second threshold is not calculated from the data, and we simply take two fixed thresholds, the error scale is still reasonably small. This is the last case shown in Figure 1 and the case used in (Rohr et al. 2022; Koch et al. 2023) with T1 being the 0.6^th^ quantile and T2 being 0.8^th^ quantile of the absolute amplitudes of the data. The mean error is then slightly bigger with 0.012 (1.2%) or 110 s.

# The Effect of the medication on the parameter choice

In the paper we use a parameter search to find a good choice of the corresponding parameter for each algorithm. We use an inverted cross-validation scheme to choose the parameter on a subset of all patients and evaluate on the rest of the patients. We stratified the data for the existence of burst suppression to avoid the parameter being chosen on a biased data set.

We also stratified for the maintenance medication and chose the parameters for sevoflurane and propofol separately. The separate parameter choice improved the error for both methods. It is notable that the error for the sevoflurane patients is smaller than for the TIVA patients (Figure 2). The thresholds chosen for the sevoflurane group were generally smaller than for the TIVA group.

# Figures

##
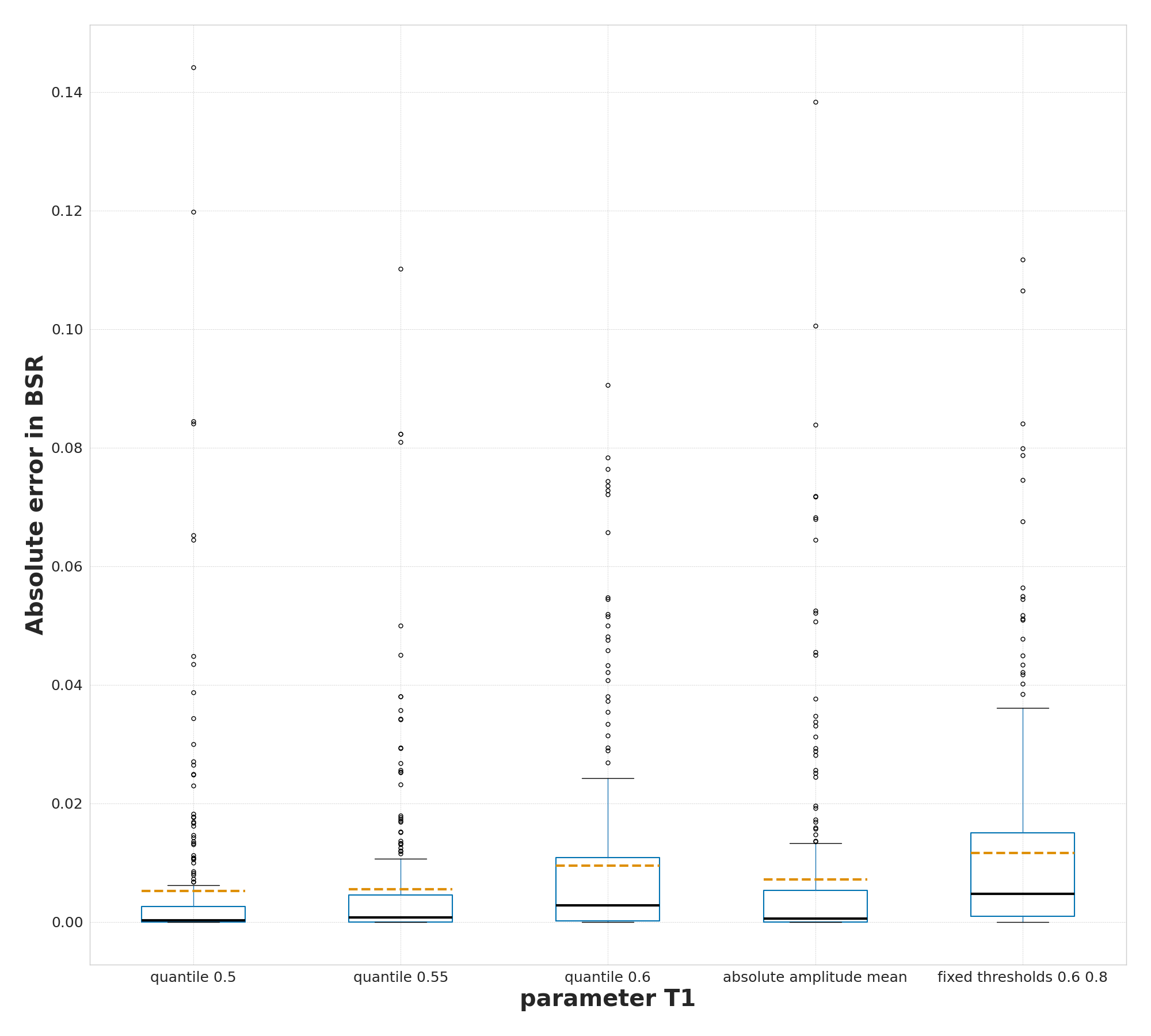
Supplement Figure 1

Supplement Figure 1 shows the absolute BSR error for Algorithm 1 dependent on the threshold parameter T1. The last case shown is different from the others, because both possible thresholds are fixed to 0.6^th^ quantile and 0.8^th^ quantile.

##
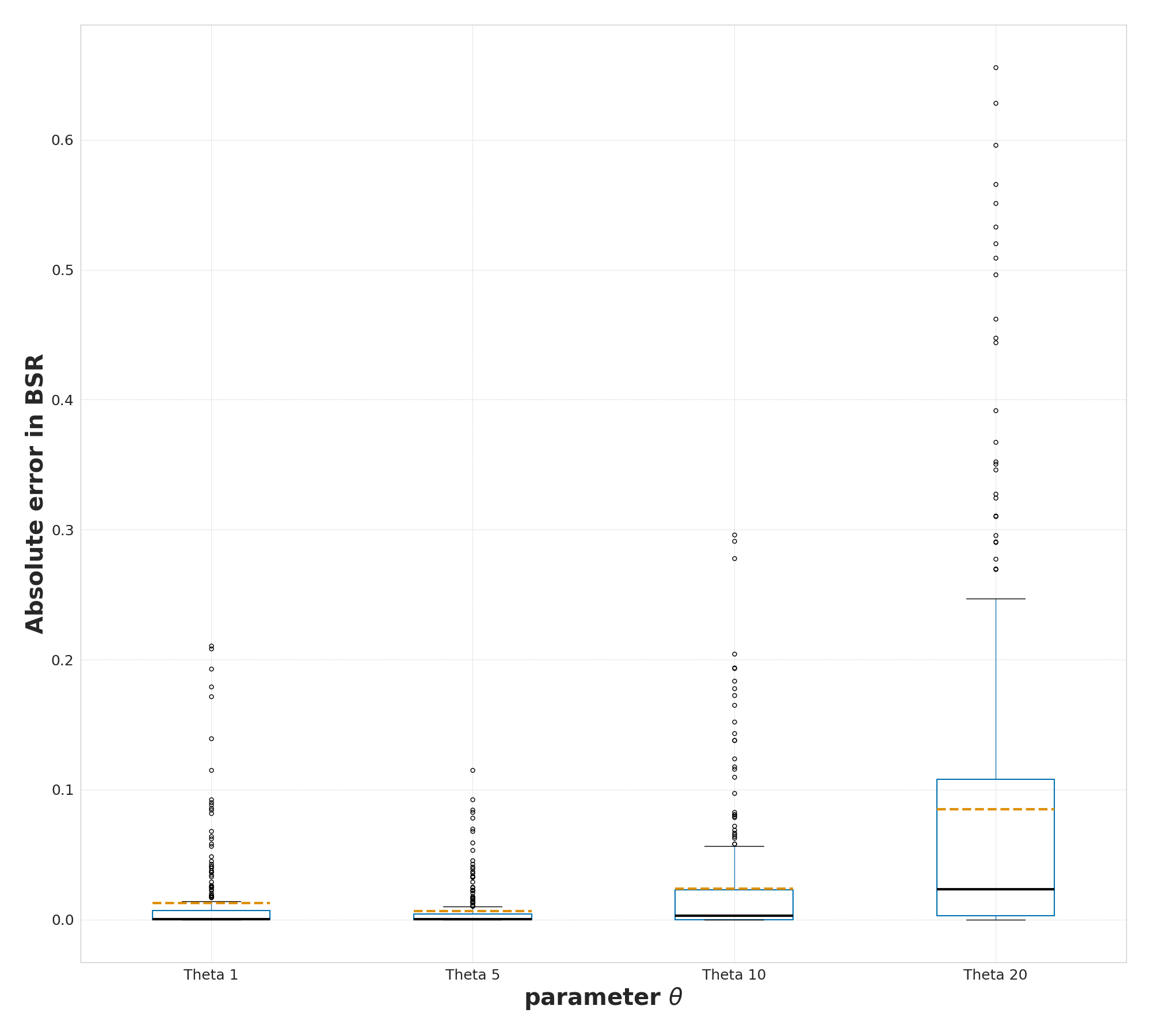
Supplement Figure 2

Supplement Figure 2 shows the absolute BSR error for Algorithm 2 dependent on the threshold parameter θ

## Supplement Figure 3


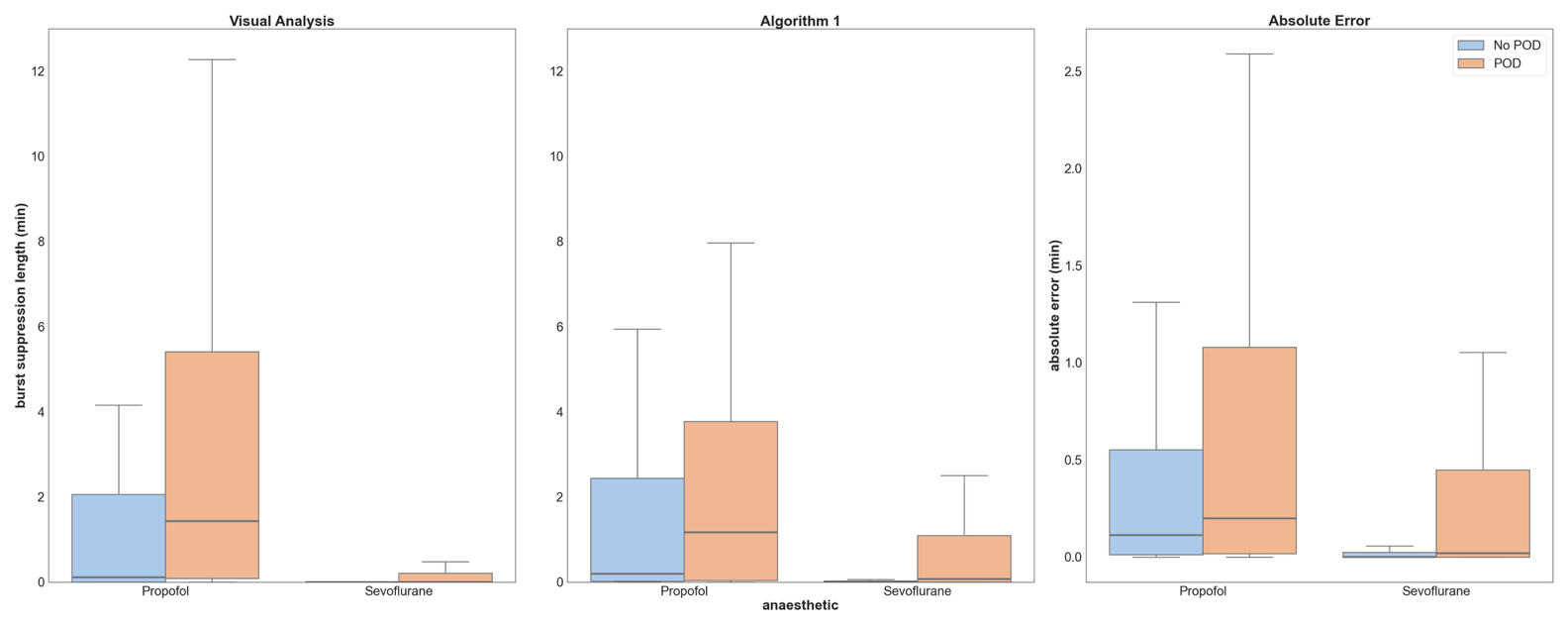


Supplement Figure 3 shows intraoperative burst suppression duration in propofol and sevoflurane patients, differentiated by POD and No POD, with the ordinate on a minute scale. The visual analysis is compared to the algorithm used for the BS detection. The median error for propofol is 11.9 s for POD and 6.7 s for No POD. For sevoflurane the median error is 1.2 s for POD and 0.0 s for No POD.

# Logistic Regression Results

Overview of Statistical Modelling Approach

We employed multiple logistic regression modelling strategies to comprehensively examine the relationships between burst suppression patterns, anesthetic agents, and postoperative delirium while addressing the methodological challenges posed by the highly skewed distribution of burst suppression data with substantial zero-inflation.

Model Selection Rationale

Given that 49% of patients had zero burst suppression duration, we implemented several complementary approaches:

- Continuous BSR Model: Using burst suppression ratio as a continuous predictor
- Raw Duration Model: Using raw burst suppression duration in seconds
- Binary Threshold Model: Modelling the presence/absence of any burst suppression
- Two-part Model: Combining binary indicators with log-transformed duration using offset methodology
- Sensitivity Analyses: Restricting analyses to patients with measurable burst suppression
- Subgroup Analyses: Separate models for each anesthetic agent

**Model 1: Main Analysis - Burst Suppression Ratio (BSR)**

This model used the burst suppression ratio (BSR) as the primary burst suppression measure.

Model Specifications:

- Sample size: 262 patients
- Pseudo R-squared: 0.0589
- Log-likelihood: -162.51
- Model p-value: 0.002

Suppl Table 1: Main BSR Model Results

| Variable | Coefficient | Std Error | z-value | p-value | 95% CI Lower | 95% CI Upper | Odds Ratio | OR 95% CI |
| --- | --- | --- | --- | --- | --- | --- | --- | --- |
| Constant | -1.989 | 2.246 | -0.885 | 0.376 | -6.392 | 2.414 | 0.137 | 0.002-2.84 |
| ASA Score | 0.109 | 0.234 | 0.466 | 0.641 | -0.349 | 0.567 | 1.115 | 0.705-1.76 |
| Sevoflurane vs Propofol | 0.789 | 0.281 | 2.805 | 0.005 | 0.238 | 1.340 | 2.200 | 1.27-3.81 |
| Age (years) | 0.001 | 0.028 | 0.027 | 0.978 | -0.053 | 0.055 | 1.001 | 0.948-1.06 |
| Surgery Duration | 0.287 | 0.085 | 3.382 | 0.001 | 0.121 | 0.453 | 1.332 | 1.13-1.57 |
| BSR | 0.039 | 0.034 | 1.132 | 0.257 | -0.028 | 0.106 | 1.040 | 0.972-1.11 |
| PACU/ICU vs Recovery | -0.272 | 0.317 | -0.859 | 0.390 | -0.894 | 0.349 | 0.762 | 0.409-1.42 |

**Model 2: Raw Burst Suppression Duration**

This model used raw burst suppression duration in seconds as the continuous predictor.

Model Specifications:

- Sample size: 262 patients
- Pseudo R-squared: 0.0567
- Log-likelihood: -162.90

Suppl Table 2: Raw Duration Model Results

| Variable | Coefficient | Std Error | z-value | p-value | 95% CI Lower | 95% CI Upper | Odds Ratio | OR 95% CI |
| --- | --- | --- | --- | --- | --- | --- | --- | --- |
| Constant | -2.025 | 2.242 | -0.903 | 0.367 | -6.420 | 2.370 | 0.132 | 0.002-2.91 |
| ASA Score | 0.118 | 0.233 | 0.506 | 0.613 | -0.339 | 0.575 | 1.125 | 0.712-1.78 |
| Sevoflurane vs Propofol | 0.757 | 0.280 | 2.705 | 0.007 | 0.209 | 1.306 | 2.133 | 1.23-3.70 |
| Age (years) | 0.002 | 0.027 | 0.069 | 0.945 | -0.052 | 0.056 | 1.002 | 0.949-1.06 |
| Surgery Duration | 0.274 | 0.084 | 3.249 | 0.001 | 0.109 | 0.439 | 1.315 | 1.12-1.55 |
| BS Duration (seconds) | 0.0002 | 0.0003 | 0.682 | 0.495 | -0.0004 | 0.0008 | 1.0002 | 0.9996-1.0008 |
| PACU/ICU vs Recovery | -0.267 | 0.317 | -0.842 | 0.400 | -0.889 | 0.355 | 0.766 | 0.411-1.43 |

**Model 3: Binary Burst Suppression Model**

This model used binary presence/absence of burst suppression and demonstrated the best model fit.

Model Specifications:

- Sample size: 262 patients
- Pseudo R-squared: 0.0922
- Log-likelihood: -156.77

Suppl Table 3: Binary Burst Suppression Model Results

| Variable | Coefficient | Std Error | z-value | p-value | 95% CI Lower | 95% CI Upper | Odds Ratio | OR 95% CI |
| --- | --- | --- | --- | --- | --- | --- | --- | --- |
| Constant | -2.101 | 2.294 | -0.916 | 0.360 | -6.597 | 2.395 | 0.122 | 0.001-2.90 |
| ASA Score | 0.019 | 0.242 | 0.077 | 0.939 | -0.455 | 0.493 | 1.019 | 0.634-1.64 |
| Sevoflurane vs Propofol | 1.186 | 0.318 | 3.727 | <0.001 | 0.562 | 1.810 | 3.274 | 1.75-6.13 |
| Age (years) | -0.003 | 0.028 | -0.097 | 0.923 | -0.057 | 0.052 | 0.997 | 0.944-1.05 |
| Surgery Duration | 0.242 | 0.085 | 2.829 | 0.005 | 0.074 | 0.409 | 1.273 | 1.08-1.51 |
| Any Burst Suppression | 1.096 | 0.318 | 3.449 | 0.001 | 0.473 | 1.719 | 2.992 | 1.60-5.58 |
| PACU/ICU vs Recovery | -0.237 | 0.320 | -0.739 | 0.460 | -0.865 | 0.391 | 0.789 | 0.421-1.48 |

**Model 4: Two-Part Model with Log Transformation**

This model combined binary burst suppression indicators with log-transformed continuous duration using offset methodology.

Model Specifications:

- Sample size: 262 patients
- Pseudo R-squared: 0.1041
- Log-likelihood: -154.71

Suppl Table 4 Two-Part Model Results

| Variable | Coefficient | Std Error | z-value | p-value | 95% CI Lower | 95% CI Upper | Odds Ratio | OR 95% CI |
| --- | --- | --- | --- | --- | --- | --- | --- | --- |
| Constant | -1.891 | 2.280 | -0.829 | 0.407 | -6.359 | 2.577 | 0.151 | 0.002-3.18 |
| ASA Score | 0.087 | 0.244 | 0.356 | 0.722 | -0.392 | 0.566 | 1.091 | 0.676-1.76 |
| Sevoflurane vs Propofol | 1.190 | 0.327 | 3.639 | <0.001 | 0.549 | 1.830 | 3.286 | 1.73-6.24 |
| Age (years) | -0.004 | 0.028 | -0.156 | 0.876 | -0.059 | 0.050 | 0.996 | 0.943-1.05 |
| Log Surgery Duration | 0.718 | 0.218 | 3.300 | 0.001 | 0.292 | 1.144 | 2.050 | 1.34-3.14 |
| Any Burst Suppression | 0.810 | 0.551 | 1.469 | 0.142 | -0.271 | 1.890 | 2.247 | 0.763-6.62 |
| Log BS (offset=0.5) | 0.043 | 0.100 | 0.433 | 0.665 | -0.153 | 0.239 | 1.044 | 0.858-1.27 |
| PACU/ICU vs Recovery | -0.369 | 0.326 | -1.130 | 0.258 | -1.008 | 0.271 | 0.691 | 0.365-1.31 |

**Sensitivity Analysis: Patients with Measurable Burst Suppression Only**

**Model 5: Raw Duration (BS > 0 only)**

Model Specifications:

- Sample size: 130 patients (with measurable BS)
- Pseudo R-squared: 0.1038

Suppl Table 5 BS-Positive Patients - Raw Duration

| Variable | Coefficient | Std Error | z-value | p-value | 95% CI Lower | 95% CI Upper | Odds Ratio | OR 95% CI |
| --- | --- | --- | --- | --- | --- | --- | --- | --- |
| Constant | -1.298 | 3.309 | -0.392 | 0.695 | -7.783 | 5.188 | 0.273 | 0.0004-178 |
| ASA Score | 0.102 | 0.356 | 0.286 | 0.775 | -0.596 | 0.799 | 1.107 | 0.551-2.22 |
| Sevoflurane vs Propofol | 1.704 | 0.447 | 3.810 | <0.001 | 0.827 | 2.580 | 5.495 | 2.29-13.2 |
| Age (years) | -0.002 | 0.040 | -0.046 | 0.963 | -0.080 | 0.076 | 0.998 | 0.923-1.08 |
| Surgery Duration | 0.158 | 0.111 | 1.433 | 0.152 | -0.058 | 0.375 | 1.172 | 0.944-1.45 |
| BS Duration (seconds) | 0.00004 | 0.0004 | 0.114 | 0.909 | -0.0007 | 0.0008 | 1.00004 | 0.9993-1.0008 |

**Subgroup Analyses by Anesthetic Agent**

**Model 6: Sevoflurane Group Only**

Model Specifications:

- Sample size: 123 patients
- Pseudo R-squared: 0.1658
- Model highly significant (p < 0.001)

Suppl Table 6 Sevoflurane Subgroup Analysis

| Variable | Coefficient | Std Error | z-value | p-value | 95% CI Lower | 95% CI Upper | Odds Ratio | OR 95% CI |
| --- | --- | --- | --- | --- | --- | --- | --- | --- |
| Constant | -4.711 | 3.441 | -1.369 | 0.171 | -11.455 | 2.034 | 0.009 | <0.001-7.65 |
| ASA Score | -0.186 | 0.356 | -0.521 | 0.602 | -0.883 | 0.512 | 0.831 | 0.414-1.67 |
| Age (years) | 0.045 | 0.042 | 1.082 | 0.279 | -0.037 | 0.127 | 1.046 | 0.964-1.14 |
| Surgery Duration | 0.345 | 0.116 | 2.964 | 0.003 | 0.117 | 0.572 | 1.411 | 1.12-1.77 |
| Any Burst Suppression | 1.790 | 0.463 | 3.862 | <0.001 | 0.881 | 2.698 | 5.984 | 2.41-14.9 |

**Model 7: Propofol Group Only**

Model Specifications:

- Sample size: 139 patients
- Pseudo R-squared: 0.0337
- Model not significant (p = 0.220)

Suppl Table 7: Propofol Subgroup Analysis

| Variable | Coefficient | Std Error | z-value | p-value | 95% CI Lower | 95% CI Upper | Odds Ratio | OR 95% CI |
| --- | --- | --- | --- | --- | --- | --- | --- | --- |
| Constant | 0.959 | 3.269 | 0.293 | 0.769 | -5.449 | 7.367 | 2.609 | 0.004-1594 |
| ASA Score | 0.152 | 0.334 | 0.455 | 0.649 | -0.502 | 0.806 | 1.164 | 0.605-2.24 |
| Age (years) | -0.038 | 0.041 | -0.939 | 0.348 | -0.118 | 0.041 | 0.963 | 0.889-1.04 |
| Surgery Duration | 0.125 | 0.100 | 1.252 | 0.211 | -0.071 | 0.321 | 1.133 | 0.931-1.38 |
| Any Burst Suppression | 0.539 | 0.446 | 1.209 | 0.227 | -0.335 | 1.413 | 1.715 | 0.715-4.11 |

**Model 8 Interaction Model**

Model Specifications:

- Sample size: 262 patients
- Pseudo R-squared: 0.1074
- Log-likelihood: -154.12
- Model p-value: <0.001

| Suppl Table 8 Interaction Model Results | | | | | | | | |
| --- | --- | --- | --- | --- | --- | --- | --- | --- |
| Variable | Coefficient | Std Error | z-value | p-value | 95% CI Lower | 95% CI Upper | Odds Ratio | OR 95% CI |
| Constant | -2.375 | 2.347 | -1.012 | 0.312 | -6.975 | 2.225 | 0.093 | 0.001-9.26 |
| ASA Score | 0.039 | 0.244 | 0.160 | 0.873 | -0.439 | 0.517 | 1.04 | 0.64-1.68 |
| Sevoflurane vs Propofol | 0.491 | 0.443 | 1.107 | 0.268 | -0.377 | 1.359 | 1.63 | 0.69-3.89 |
| Age (years) | 0.006 | 0.029 | 0.213 | 0.832 | -0.051 | 0.063 | 1.01 | 0.95-1.06 |
| Surgery Duration | 0.287 | 0.090 | 3.181 | 0.001 | 0.111 | 0.464 | 1.33 | 1.12-1.59 |
| Any Burst Suppression | 0.404 | 0.440 | 0.918 | 0.359 | -0.458 | 1.267 | 1.50 | 0.63-3.55 |
| **Sevoflurane × BS Interaction** | **1.335** | **0.628** | **2.127** | **0.033** | **0.104** | **2.565** | **3.80** | **1.11-13.01** |
| PACU/ICU vs Recovery | -0.445 | 0.501 | -0.888 | 0.375 | -1.427 | 0.537 | 0.64 | 0.24-1.71 |

**Clinical Risk Stratification Based on an Interaction Model:**

For the clinical Risk Stratification, an interaction Model was employed:

logit(POD) = β₀ + β₁(Sevoflurane) + β₂(Any_BS) + β₃(Sevoflurane × Any_BS)

β₁ = 0.4910 (main effect of sevoflurane)

β₂ = 0.4044 (main effect of burst suppression)

β₃ = 1.3346 (interaction effect)

The significant interaction term (OR = 3.80, p = 0.033) reveals risk profiles:

1. Propofol + No BS (Reference)
   1. Linear predictor: 0 (reference group)
   2. Odds Ratio: = 1.00
2. Propofol + BS
   1. Linear predictor: 0 + 0.4044 + 0 = 0.4044
   2. Odds Ratio: e^0.4044 = 1.50 (95% CI: 0.63–3.55)
3. Sevoflurane + No BS
   1. Linear predictor: 0.4910 + 0 + 0 = 0.4910
   2. Odds Ratio: e^0.4910 = 1.63 (95% CI: 0.69–3.89)
4. Sevoflurane + BS
   1. Linear predictor: 0.4910 + 0.4044 + 1.3346 = 2.230
   2. Odds Ratio: e^2.230 = 9.30 (95% CI: 1.64–52.76)

The interaction term in our model, with a coefficient of 1.3346, indicates that burst suppression is approximately e^1.3346=3.80 times more deleterious in patients receiving sevoflurane than in those receiving propofol. When both sevoflurane administration and burst suppression occur together, the combined effect corresponds to a 9.30-fold increase in the odds of developing postoperative delirium compared to the reference scenario of propofol without any burst suppression. Moreover, the risk associated with burst suppression under sevoflurane is 6.2 times greater than the risk of burst suppression under propofol alone (9.30 divided by 1.50). These findings demonstrate that burst suppression and sevoflurane interact in a synergistic manner to markedly elevate the risk of postoperative delirium, far exceeding what would be expected from the sum of their individual effects.

# Dose-Response Interaction

In our dose-response analysis, sevoflurane exhibited no discernible dose-dependent effect on postoperative delirium risk, with the end-tidal concentration coefficient near zero (β = -0.0053, z = -0.011, p = 0.991) and confidence intervals spanning a wide range that included zero (-0.906 to 0.896). For propofol also no dose-response relationship was observed (β = -0.3167 mg/kg/h, z = -1.755, p = 0.079), with the 95% confidence interval narrowly including zero (-0.670 to 0.037).

| Model 6a Sevoflurane | | | | | | |
| --- | --- | --- | --- | --- | --- | --- |
| Variable | Coefficient | Std Error | z-value | p-value | 95% CI Lower | 95% CI Upper |
| Constant | -0.1917 | 0.804 | -0.238 | 0.812 | -1.768 | 1.385 |
| Sevo end exp% | -0.0053 | 0.460 | -0.011 | 0.991 | -0.906 | 0.896 |

| Model 7a Propofol | | | | | | |
| --- | --- | --- | --- | --- | --- | --- |
| Variable | Coefficient | Std Error | z-value | p-value | 95% CI Lower | 95% CI Upper |
| Constant | -0.1917 | 0.804 | -0.238 | 0.812 | -1.768 | 1.385 |
| Propofol mg/kg/h | -0.3167 | 0.180 | -1.755 | 0.079 | -0.670 | 0.037 |

# Sensitivity Analysis

To assess whether a composite measure of BS burden provides additional predictive value for POD beyond anesthetic type, we conducted two logistic regression analyses. The first model included BS duration and BSR as independent predictors. Neither variable showed a significant association with POD (p=0.764 and p=0.496, respectively; pseudo R²=0.003).

The second, fully adjusted model incorporated anesthetic type (sevoflurane vs. propofol), age, ASA score, and operation duration alongside BS metrics. In this model, anesthetic type remained the only significant predictor of POD (p<0.001), while BS duration (p=0.936) and BSR (p=0.989) remained non-significant.

Overall, these findings indicate that a composite measure of BS burden does not improve the prediction of POD risk beyond anesthetic type. The anesthetic agent itself appears to be the dominant explanatory factor among the studied variables.

| Table: Model 1 - Burst-Suppression | | | | | | |
| --- | --- | --- | --- | --- | --- | --- |
| Variable | Coefficient | Std Error | z-value | P-value | 95% CI Lower | 95% CI Upper |
| Constant | -0.1209 | 0.202 | -0.598 | 0.550 | -0.517 | 0.275 |
| BS Duration (sec) | 0.0002 | 0.001 | 0.300 | 0.764 | -0.001 | 0.001 |
| BSR (Burst Suppression Ratio) | -0.0382 | 0.056 | -0.681 | 0.496 | -0.148 | 0.072 |

Model Statistics: Pseudo R² = 0.003 | Log-Likelihood = -89.248 | LLR p-value = 0.7357 | N = 130

| Table: Model 2 - Full Model with Anesthetic Type and Confounders | | | | | | |
| --- | --- | --- | --- | --- | --- | --- |
| Variable | Coefficient | Std Error | z-value | P-value | 95% CI Lower | 95% CI Upper |
| Constant | -1.2955 | 3.313 | -0.391 | 0.696 | -7.788 | 5.197 |
| ASA Score | 0.1017 | 0.356 | 0.286 | 0.775 | -0.596 | 0.799 |
| Sevoflurane | 1.7034 | 0.448 | 3.804 | <0.001** | 0.826 | 2.581 |
| Age (years) | -0.0018 | 0.040 | -0.046 | 0.963 | -0.080 | 0.076 |
| Operation Duration (hours) | 0.1577 | 0.123 | 1.284 | 0.199 | -0.083 | 0.398 |
| BS Duration (sec) | 0.00005 | 0.001 | 0.080 | 0.936 | -0.001 | 0.001 |
| BSR (Burst Suppression Ratio) | -0.0009 | 0.063 | -0.014 | 0.989 | -0.125 | 0.123 |

Model Statistics: Pseudo R² = 0.104 | Log-Likelihood = -80.263 | LLR p-value = 0.005 | N = 130

# Tables

| Suppl. Table 9 Burst Suppression Duration Visual Analysis | | | |
| --- | --- | --- | --- |
| Suppl Table 1 | Median | 25^th^ Percentile | 75^th^ Percentile |
| All Patients (N = 265) | 0.00 | 0.00 | 80.86 |
| POD (N = 98) | 6.95 | 0.00 | 92.97 |
| NoPOD (N = 167) | 0.00 | 0.00 | 48.99 |
| Propofol (N = 139) | 20.64 | 0.00 | 151.71 |
| POD (N = 42) | 86.01 | 5.29 | 334.79 |
| NoPOD (N = 97) | 6.95 | 0.00 | 123.85 |
| Sevoflurane (N=126) | 0.00 | 0.00 | 4.94 |
| POD (N = 56) | 0.00 | 0.00 | 13.29 |
| NoPOD (N = 70) | 0.00 | 0.00 | 0.00 |
|  | | | |

This table reports the burst suppression duration for all patients combined, stratified by the presence or absence of postoperative delirium. Additionally, patients are subdivided by propofol and sevoflurane anesthesia and further stratified by delirium status within each anesthesia category. Values are reported as medians with the corresponding 25th and 75th percentiles

| Suppl. Table 10 Baseline Characteristics according to anesthetic protocol | | | |
| --- | --- | --- | --- |
|  |  | **Propofol**  **maintenance** | **Sevoflurane**  **maintenance** |
| Age (years, mean, 95%-CI) |  | 77 (76-78) | 77 (77-78) |
| Sex (N, %) | male | 37 (26.6) | 78 (61.9) |
|  | female | 102 (73.4) | 48 (38.1) |
| BMI (kg/m², mean, 95%-CI) |  | 25 (25-26) | 25 (23-27) |
| ASA-Score (N, %) | 1 | 5 (3.6) | 3 (2.4) |
|  | 2 | 68 (48.9) | 49 (38.9) |
|  | 3 | 65 (46.8) | 70 (55.6) |
|  | 4 | 1 (0.7) | 4 (3.2) |
| Highest education (N, %) | 9th grade leaving certificate | 16 (13.6) | 20 (17.7) |
|  | intermediate school leaving certificate | 39 (33.1) | 35 (31.0) |
|  | higher education entrance qualification, master craftsmen | 24 (20.3) | 20 (17.7) |
|  | Bachelor´s degree | 9 (7.6) | 5 (4.4) |
|  | Master´s degree, university diploma or higher | 30 (25.4) | 33(29.2) |
| Surgery Type (N, %) | Ophthalmology | 1 (0.7) | 0 |
|  | Traumatology/orthopedics | 38 (27.3) | 27 (21.4) |
|  | ENT/oral and maxillofacial surgery | 3 (2.2) | 3 (2.4) |
|  | Thoracic surgery | 11 (7.9) | 0 |
|  | General surgery | 50 (36.0) | 70 (55.6) |
|  | Gynecology | 31 (22.3) | 13 (10.3) |
|  | other | 5 (3.6) | 13 (10.3) |
| Premedication (N, %) | no premedication | 133 (95.7) | 122 (96.8) |
|  | Midazolam p.o | 6 (4.3) | 4 (3.2) |
|  | Midazolam i.v. | 0 | 0 |
| Surgery duration (min, mean, 95%-CI) |  | 173 (153-192) | 162 (142-181) |
| Postoperative Transfer (N, %) | Recovery room | 72 (51.8) | 57 (45.2) |
|  | ICU | 51 (36.7) | 57 (45.2) |
|  | PACU | 15 (10.8) | 12 (9.5) |
| Anesthetic Dose | Propofol mg/kg/h (standard deviation) | 5.58 (1.083) |  |
|  | Sevoflurane (End tidal Sev) (standard deviation) |  | 1.7 (0.402) |

# 8. Tables for Algorithm Performance

| Suppl. Table 11 Algorithm 1 results | | | | | |
| --- | --- | --- | --- | --- | --- |
| A1 | Mean (s) | Std (s) | Median (s) | IQR 25^th^ | IQR 75^th^ |
| TIVA NoPOD | | | | | |
| BS Duration | 198.3439 | 498.6508 | 11.8437 | 1.291 | 146.2051 |
| BSR | 0.0057 | 0.0113 | 0.001 | 0.0001 | 0.0053 |
| Error | 50.5669 | 99.581 | 6.7358 | 0.6567 | 33.1867 |
| TIVA POD | | | | | |
| BS Duration | 228.3986 | 447.5014 | 70.2062 | 2.1877 | 226.3212 |
| BSR | 0.0089 | 0.0186 | 0.0013 | 0.0001 | 0.0059 |
| Error | 87.3641 | 167.4869 | 11.9394 | 1.0226 | 64.7767 |
| Sevoflurane NoPOD | | | | | |
| BS Duration | 24.0728 | 76.9251 | 0.0 | 0.0 | 2.0544 |
| BSR | 0.0025 | 0.011 | 0.0 | 0.0 | 0.0002 |
| Error | 12.2763 | 49.3591 | 0.0 | 0.0 | 1.4559 |
| Sevoflurane POD | | | | | |
| BS Duration | 106.8041 | 370.2917 | 4.1818 | 0.0 | 65.7522 |
| BSR | 0.0049 | 0.0129 | 0.0001 | 0.0 | 0.0029 |
| Error | 59.2517 | 204.6115 | 1.1566 | 0.0 | 26.9436 |

BS Duration = Burst Suppression Duration, BSR = Burst Suppression Ratio

This table summarizes the performance metrics of algorithm 1

| Suppl Table 12 Algorithm 2 results | | | | | |
| --- | --- | --- | --- | --- | --- |
| A2 | Mean (s) | Std (s) | Median (s) | IQR 25^th^ | IQR 75^th^ |
| TIVA NoPOD | | | | | |
| BS Duration | 264.6938 | 695.0663 | 22.3515 | 0.4547 | 177.3411 |
| BSR | 0.0084 | 0.016 | 0.0015 | 0.0 | 0.01 |
| Error | 88.8403 | 215.6971 | 8.5104 | 0.0112 | 62.9323 |
| TIVA POD | | | | | |
| BS Duration | 307.7001 | 653.2064 | 77.1215 | 4.3951 | 275.6831 |
| BSR | 0.0133 | 0.0255 | 0.0013 | 0.0001 | 0.0105 |
| Error | 140.7292 | 281.3663 | 13.1133 | 1.3446 | 118.5072 |
| Sevoflurane NoPOD | | | | | |
| BS Duration | 23.2552 | 78.1108 | 0.0 | 0.0 | 0.0 |
| BSR | 0.0026 | 0.0115 | 0.0 | 0.0 | 0.0 |
| Error | 12.1912 | 49.2945 | 0.0 | 0.0 | 0.1586 |
| Sevoflurane POD | | | | | |
| BS Duration | 86.2125 | 300.4877 | 4.8469 | 0.0 | 65.8251 |
| BSR | 0.0035 | 0.0073 | 0.0003 | 0.0 | 0.004 |
| Error | 39.2768 | 114.3623 | 3.4208 | 0.0 | 42.9366 |

BS Duration = Burst Suppression Duration, BSR = Burst Suppression Ratio

This table summarizes the performance metrics of algorithm 2
